# Retroform Cervical Dystonia: Target Muscle Selection and Efficacy of Botulinum Toxin Injection

**DOI:** 10.3389/fneur.2022.952456

**Published:** 2022-07-26

**Authors:** Yaowen Hu, Lizhen Pan, Junhui Su, Shuzhen Chen, Xiaolong Zhang, Yougui Pan, Lingjing Jin, Fei Teng

**Affiliations:** ^1^Department of Neurology, School of Medicine, Neurotoxin Research Center, Tongji Hospital, Tongji University, Shanghai, China; ^2^Department of Nuclear Medicine, School of Medicine, Tongji Hospital, Tongji University, Shanghai, China; ^3^Department of Neurology and Neurological Rehabilitation, School of Medicine, Shanghai Yangzhi Rehabilitation Hospital, Tongji University, Shanghai, China

**Keywords:** retrocaput, retrocollis, cervical dystonia, botulinum toxin, single-photon emission computed tomography

## Abstract

**Introduction:**

Retroform cervical dystonia (RCD), which includes retrocaput and retrocollis, is a rare form of cervical dystonia. Few reports have been published on RCD. The present study aimed to characterize the target muscles involved in RCD and the efficacy of botulinum toxin type A (BTX-A) injection.

**Methods:**

Patients with consecutive cervical dystonia with RCD as the most problematic feature were retrospectively analyzed over a 10-year period. Target muscles were screened and confirmed based on clinical evaluation, single-photon emission computed tomography, and electromyography. In addition, efficacy and adverse events following BTX-A injection in patients with RCD were evaluated.

**Results:**

A total of 34 patients with RCD were included, 18 of whom presented with retrocaput and 16 with retrocollis. The most frequently injected muscles in RCD were splenius capitis (SPCa, 97.1%) and semispinalis capitis (SSCa, 97.1%), followed by levator scapulae (LS, 50.0%), rectus capitis posterior major (RCPM, 47.1%), trapezius (TPZ, 41.2%), and sternocleidomastoid muscle (SCM, 41.2%). Besides cervical muscles, the erector spinae was also injected in 17.6% of patients. Most muscles were predominantly bilaterally injected. The injection schemes of retrocaput and retrocollis were similar, possibly because in patients with retrocollis, retrocaput was often combined. BTX-A injection achieved a satisfactory therapeutic effect in RCD, with an average symptom relief rate of 69.0 ± 16.7%. Mild dysphagia (17.6%) and posterior cervical muscle weakness (17.6%) were the most common adverse events.

**Conclusion:**

SPCa, SSCa, LS, RCPM, LS, and SCM were commonly and often bilaterally injected in RCD. Patients with RCD could achieve satisfactory symptom relief after BTX-A injection.

## Introduction

Head and neck extension, which is defined as retrocollis in traditional classification, is a rare form of cervical dystonia (CD). At present, the new Col-Cap (Col = collis, Cap = caput) concept specifically defined head extension as retrocaput and neck extension as retrocollis (1). To avoid confusion with the old classification, we called retrocaput and retrocollis together as retroform CD (RCD).

In a study including over 1,000 patients with CD, the proportion of RCD was only 5.3% (2). In a recent cohort study based on the new concept, Jost et al. reported that 4.6% of the patients with CD presented with retrocaput and 2.9% with retrocollis (3). Probably due to its relatively low incidence, scarce reports have been focused on RCD. Furthermore, RCD has been frequently excluded from CD clinical trials with different formulations of botulinum toxin type A (BTX-A) (4, 5), which could have possibly been because the extension of the head and neck could be secondary to tardive movement disorder or other neurodegenerative movement disorders such as progressive supranuclear palsy (6). A study published in 2008 was the only large RCD cohort assessed. However, this earlier investigation focused mainly on the clinical characteristics of RCD, whereas the discussion on the target dystonic muscle involved in RCD was insufficient (6).

Dressler et al. suggested that head extension originates from bilateral activation of splenius capitis (SPCa) and the deep posterior neck muscles, whereas neck extension is elicited by bilateral activation of the trapezius (TPZ) and semispinalis capitis (SSCa) (7). In our center, we developed ^99m^Technetium-sestamibi single-photon emission computed tomography (^99m^Tc-MIBI SPECT/CT) as a new method for screening affected muscles in CD, which has been proven to be a reliable method for identifying dystonic muscles (8, 9). Combining the screening of SPECT/CT with electromyography (EMG) detection, we detected a wider range of muscles in RCD.

Based on the above background, the goal of this retrospective study was to characterize the target muscles involved in RCD as well as their responses to BTX-A injection.

## Materials and Methods

### Patients

Patients with consecutive CD admitted to the Movement Disorders Center of Tongji University affiliated with Tongji Hospital (Shanghai, China) from January 2012 to February 2022 were analyzed. The study was approved by the Institutional Research Ethics Committee of Tongji Hospital affiliated with Tongji University. Informed written patient consent was obtained from every patient before the procedure.

The diagnosis of RCD was made if the patient with CD exhibited any degree of abnormal head or neck extension over 10°. For patients who combined other cervical movement patterns or dystonic movements in other parts of the body, only those who had RCD as their most problematic feature were included. Patients who had been diagnosed with tardive movement disorder, Parkinson's disease, or other neurodegenerative movement disorders were excluded.

### RCD Classification

**Figure 1:**
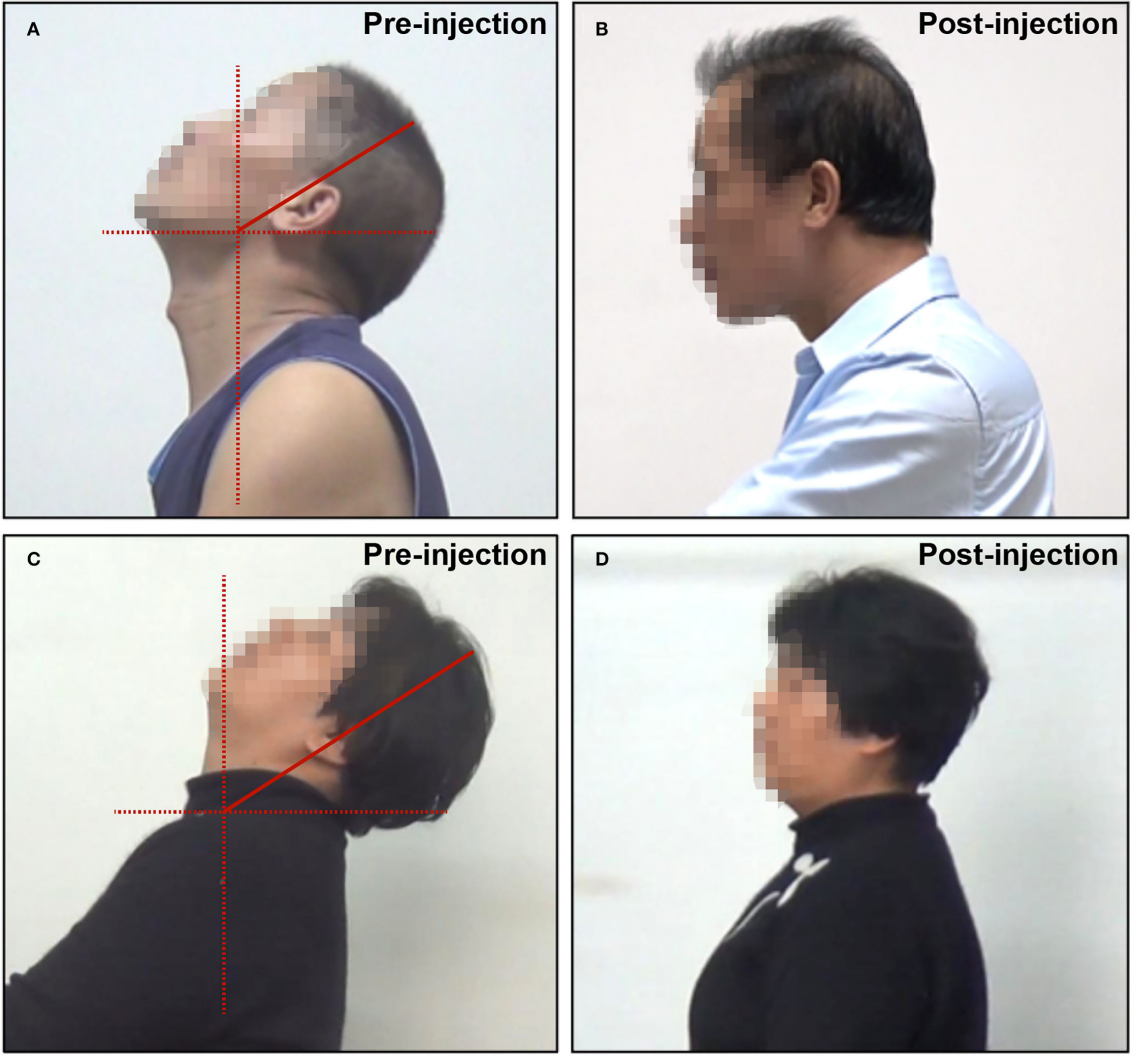
Representative pictures of patients with RCD. **(A)** Retrocaput before BTX-A injection; **(B)** retrocaput after BTX-A injection; **(C)** retrocollis before BTX-A injection; and **(D)** retrocollis after BTX-A injection.

Videos and still pictures of the sagittal aspect of the patient were carefully viewed and examined. Retrocaput was confirmed by observing the abnormal angle between the head and neck, with the angle between the cervical and thoracic spine being normal (Figure 1A). Retrocollis was diagnosed by observing the abnormal angle between the cervical and thoracic spine (Figure 1C).

### Target Muscle Selection Strategy and BTX-A Injection

Target muscle selection and BTX-A injection were performed by experienced neurologists. Target muscles were firstly selected by clinical evaluation (movement pattern, muscle palpation, and local pain). For 19 patients who received BTX-A injection after 2015, SPECT/CT images were also considered for target muscle selection. For SPECT/CT, ^99m^Technetium-Sestamibi (^99m^Tc-MIBI, Shanghai atom Kexing pharmaceutical Company Limited) was used as a developing agent, and Precedence SPECT (Philips, Netherlands) was used for imaging. The scan was performed 1 h after the intravenous injection of 740 MBq of ^99m^Tc-MIBI. CT tomography was performed with a slice thickness of 5 mm, pitch of 1, and a matrix of 512 × 512. Subsequently, SPECT tomography was performed with a peak acquisition energy of 140 keV, window width 20%, matrix 64 × 64, magnification 1, probe rotation of 360°, 6°/frame, 15 s/frame, and continuous 32 frames. After the acquisition, SPECT and CT images were fused. The images were observed by experienced physicians. Each muscle with increased ^99m^Tc-MIBI uptake was analyzed as previously reported (8).

All selected muscles were detected using a needle EMG (model NTS-2000, NCC Medical Co., Ltd, China) during BTX-A injection. Recordings were obtained with the patients sitting in a chair allowing their head, neck, and shoulders to assume an involuntary posture. They were instructed not to resist the abnormal posture. As previously reported, a muscle was considered dystonic if it satisfied these three criteria: (1) the EMG displayed consistent tonic or phasic patterns of discharge; (2) the amplitude of the discharge was ≥50% of the amplitude during maximal voluntary activation; and (3) the EMG discharge occurred in the presence of the patient's abnormal posture (10).

BTX-A injection was performed only in the muscles whose dystonic activity was confirmed by EMG. The dose for each individual muscle was mainly based on the severity and pattern of the disease, clinical experience, and muscle volume. The EMG discharge of the muscles as well as their radioactivity revealed by SPECT images were also considered for the determination of the BTX-A dose. The choice of BTX-A [lanbotulinumtoxinA (Hengli® Lanzhou Institute of Biological Products, Lanzhou, China) or onabotulinumtoxinA (Botox®, Abbvie, Chicago, IL, USA)] depended on the patients' will, and the dose conversion ratio of these two types of BTX-A was 1:1 (11). The efficacy of BTX-A was assessed by subjective overall symptom relief rate (SRR).

### Statistical Analysis

All statistical analyses were conducted through SPSS version 21.0 (SPSS Inc., Chicago, IL, USA). Quantitative data are expressed as mean ± standard deviation (SD). The Shapiro–Wilk test was conducted for assessing the normality of data. Normally distributed data were compared using a *t*-test, whereas data that did not meet the normal distribution criteria were compared through the Mann–Whitney U-test. Categorical variables were compared by the chi-squared test. *P* < 0.05 was considered to indicate statistically significant differences.

## Results

### Demographical and Clinical Characteristics of Patients With RCD

A total of 1,243 patients with CD admitted to our center during the period of the study were reviewed, 36 (2.9%) of whom had RCD as the predominant movement pattern. However, the clinical data of two patients were incomplete and were thus ruled out from the study. Of the 34 patients included, four had a history of neuroleptics, which included Ziprasidone, Sertraline, Olanzapine, Risperidone, Mirtazapine, and Venlafaxine.

Demographical data of the 34 patients are presented in Table 1. Of the 34 patients, 21 (61.8%) patients combined other cervical movement patterns: twelve with rotation, six with tremor, five with lateral flexion, and three with a sagittal shift. Thirteen patients (38.2%) associated dystonic movements of other areas: oromandibular dystonia in four patients, dystonia of the limb in four patients, dystonia of the trunk in three patients, and Meige syndrome in two patients. A total of seven patients received genetic testing, two of whom were found abnormal, one was with DYT1, and the other with DYT6.

**Table 1:** Demographical and clinical characteristics in patients with RCD.

**Demographical and clinical parameters**	**RCD patients (*n* = 34)**	**Retrocaput** **(*n* = 18)**	**Retrocollis (*n* = 16)**	*P* value^*^
Gender (Male/Total)	17/34 (50.0%)	8/18 (44.4%)	9/16 (56.3%)	0.492
Age (years)	49.0 ± 12.0	48.1 ± 11.7	50.0 ± 12.6	0.645
Duration of symptoms (years)	7.6 ± 6.8	7.2 ± 2.9	8.0 ± 9.6	0.330
Patients with history of BTX-A injection	11/34 (32.4%)	5/18 (27.8%)	6/16 (37.5%)	0.545
Tsui score before injection	8.8 ± 3.4	8.9 ± 3.4	8.6 ± 3.5	0.789
Combination of other cervical movement patterns	21/34 (61.8%)	13/18 (72.2%)	8/16 (50%)	0.183
Combination of dystonic movements in other areas of the body	13/34 (38.2%)	3/18 (16.7%)	10/16 (62.5%)	0.006^*^
BTX-A dose (units)	260.3 ± 80.5	241.7 ± 54.2	281.3 ± 100.1	0.266
SRR (%)	69.0 ± 16.7	70.0 ± 14.0	67.8 ± 19.7	0.710

* *The comparison was performed between patients with retrocaput and patients with retrocollis*.

*RCD, retroform cervical dystonia; BTX-A, botulinum toxin type A; SRR, subjective overall symptom relief rate*.

Twenty-seven patients received an injection of Hengli®, and seven patients received an injection of Botox®. The average BTX-A dose was 260.3 ± 80.5 units. The overall symptom relief was satisfactory, with an average SRR of 69.0 ± 16.7%. The majority of the patients (19/34, 55.9%) had an SRR ≥70% (Figures 1B,D). The SRR of 12 patients (35.3%) was between 40 and 70%, while only three patients (8.8%) had SRR <40%.

### Frequency of Injection and BTX-A Dose for Each Cervical Muscle in Patients With RCD

The frequency of injection for each muscle (the number of patients who received an injection in this muscle /total number of patients) was calculated and is listed in Table 2. The average dose for each muscle (for bilateral injection, the doses of the bilateral muscles were calculated as two muscles) and the range of the doses used for each muscle are also presented in Table 2.

**Table 2:** Frequency of injection and BTX-A dose for each cervical muscle.

**Muscle**	**Frequency of injection**			**BTX-A dose (Units)**	
	**Total**	**Bilateral injection**	**Unilateral injection**	**Average ± SD**	**Minimum-Maximum**
**All patients (n** **=** **34)**					
SPCa	97.1% (33)	94.1 % (32)	2.9% (1)	37.7 ± 19.2	12.5–75
SSCa	97.1% (33)	88.2% (30)	8.8% (3)	34.7 ±14.5	12.5–75
LS	50.0% (17)	29.4% (10)	20.6% (7)	32.4 ± 12.6	12.5–50
RCPM	47.1% (16)	44.1% (15)	2.9% (1)	19.0 ± 6.9	6.25–25
TPZ	41.2% (14)	20.6% (7)	20.6% (7)	23.8 ± 7.8	12.5–37.5
SCM	41.2% (14)	20.6% (7)	20.6% (7)	32.1 ± 16.1	12.5–62.5
ES	17.6% (6)	17.6% (6)	0	59.4 ± 49.5	12.5–150
RCPm	11.8% (4)	8.8% (3)	2.9% (1)	12.5 ± 0	12.5–12.5
LGCa	11.8% (4)	5.9% (2)	5.9% (2)	25.0 ± 7.9	12.5–37.5
Sc	8.8% (3)	0	8.8% (3)	25.0 ± 12.5	12.5–37.5
OCI	8.8% (3)	2.9% (1)	5.9% (2)	12.5 ± 0	12.5–12.5
SSCe	5.9% (2)	5.9% (2)	0	43.8 ± 7.2	37.5–50
SPCe	2.9% (1)	2.9% (1)	0	12.5 ± 0	12.5–12.5
**Retrocaput (n = 18)**					
SPCa	100% (18)	94.4% (17)	5.6% (1)	41.8 ± 21.4	12.5–75
SSCa	100% (18)	88.9% (16)	11.1% (2)	35.7 ± 15.7	12.5–75
LS	50.0% (9)	22.2% (4)	27.8% (5)	33.7 ± 10.7	12.5–50
TPZ	44.4% (8)	16.7% (3)	27.8% (5)	22.7 ± 9.4	12.5–37.5
SCM	33.3% (6)	11.1% (2)	22.2% (4)	39.1 ± 17.0	12.5–62.5
RCPM	33.3% (6)	33.3% (6)	0	19.8 ± 6.4	12.5–25
ES	11.1% (2)	11.1% (2)	0	18.8 ± 7.2	12.5–25
OCI	11.1% (2)	5.6% (1)	5.6% (1)	12.5 ± 0	12.5–12.5
Sc	11.1% (2)	0	11.1% (2)	18.8 ± 8.8	12.5–25
RCPm	5.6% (1)	5.6% (1)	0	12.5 ± 0	12.5–12.5
LGCa	5.6% (1)	5.6% (1)	0	31.3 ± 8.8	25–37.5
SSCe	0	0	0	/	/
SPCe	0	0	0	/	/
**Retrocollis (n = 16)**					
SPCa	93.8% (15)	93.8% (15)	0	32.9 ± 15.2	12.5–62.5
SSCa	93.8% (15)	87.5% (14)	6.3% (1)	33.6 ± 13.0	12.5–50
RCPM	62.5% (10)	56.3% (9)	6.3% (1)	18.4 ± 7.4	6.25–25
LS	50.0% (8)	37.5% (6)	12.5% (2)	31.3 ± 14.5	12.5–50
SCM	50.0% (8)	31.3% (5)	18.8% (3)	27.9 ± 14.6	12.5–50
TPZ	37.5% (6)	25.0% (4)	12.5% (2)	25.0 ± 5.9	12.5–37.5
ES	25.0% (4)	25.0% (4)	0	79.7 ± 49.1	25–150
RCPm	18.8% (3)	12.5% (2)	6.3% (1)	12.5 ± 0	12.5–12.5
LGCa	18.8% (3)	6.3% (1)	12.5% (2)	21.9 ± 6.3	12.5–25
SSCe	12.5% (2)	12.5% (2)	0	43.8 ± 7.2	37.5–50
SPCe	6.3% (1)	6.3% (1)	0	12.5 ± 0	12.5–12.5
Sc	6.3% (1)	0	6.3% (1)	37.5	37.5
OCI	6.3% (1)	0	6.3% (1)	12.5	12.5

*SPCa, splenius capitis; SSCa, semispinalis capitis; LS, levator scapulae; TPZ, trapezius; SCM, sternocleidomastoid; RCPM, rectus capitis posterior major; RCPm, rectus capitis posterior minor; ES, erector spinae; LGCa, longissimus capitis; Sc, scalenus; OCI, obliquus capitis inferior; SSCe, semispinalis cervicis; SPCe, splenius cervicis*.

The most frequently injected muscles were SPCa (97.1%) and SSCa (97.1%). Both of them were bilaterally injected in most of the patients (32/33 for SPCa and 30/33 for SSCa). Levator scapulae (LS, 50.0%), rectus capitis posterior major (RCPM, 47.1%), TPZ pars descendens (41.2%), and sternocleidomastoid muscle (SCM, 41.2%) were also commonly injected. For RCPM, almost all patients received a bilateral injection (15/16), while for LS (10/17), TPZ (7/14), and SCM (7/14), about half patients received bilateral injection. Erector spinae (ES, 17.6%), rectus capitis posterior minor (RCPm, 11.8%), and longissimus capitis (LGCa, 11.8%) were less commonly injected. Other muscles, including scalenus, obliquus capitis inferior (OCI), semispinalis cervicis (SSCe), and splenius cervicis (SPCe), were rarely injected.

**Figure 2:**
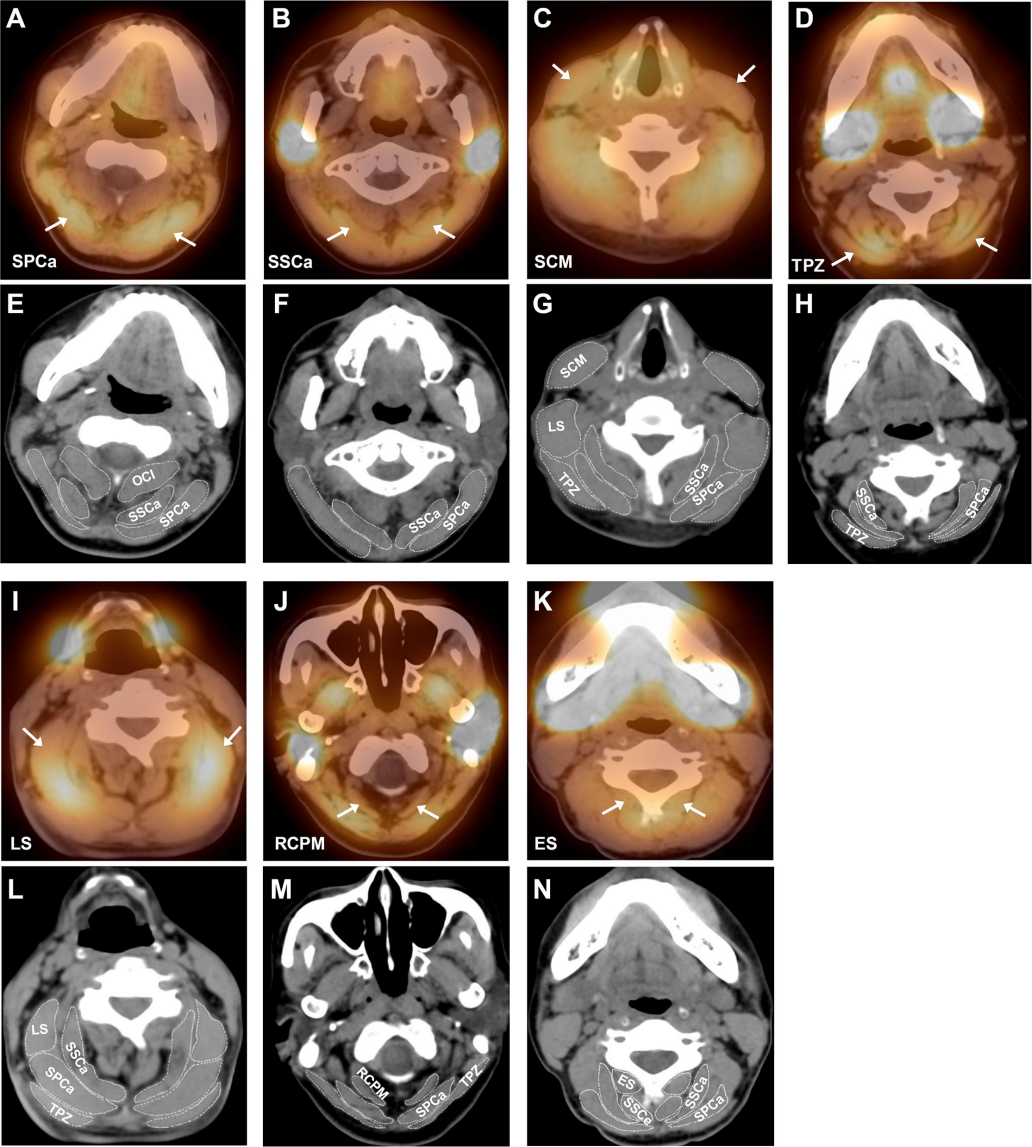
Representative SPECT/CT images of different activated cervical muscles in patients with RCD. **(A)** Bilateral activation of splenius capitis (SPCa) indicated by the white arrows; **(B)** bilateral activation of semispinalis capitis (SSCa) indicated by the white arrows; **(C)** bilateral activation of sternocleidomastoid muscle (SCM) indicated by the white arrows; **(D)** bilateral activation of trapezius (TPZ) indicated by the white arrows; **(I)** bilateral activation of levator scapulae (LS) indicated by the white arrows; **(J)** bilateral activation of rectus capitis posterior major (RCPM) indicated by the white arrows; and **(K)** bilateral activation of erector spinae (ES) indicated by the white arrows. To demonstrate the anatomy of cervical muscles more clearly, we put the CT images of the same level below each SPECT image and outlined the boundaries of related muscles. **(E–H)** are CT images of **(A–D)** respectively, and **(L–N)** are CT images of **(I–K)** respectively. OCI, obliquus capitis inferior; SSCe, semispinalis cervicis.

The SPECT/CT images (Figure 2) showed the bilateral involvement of these muscles. In order to display the activation of these muscles, we selected seven typical SPECT/CT images with bilateral muscle activation: SPCa, SSCa, SCM, TPZ, LS, RCPM, and ES.

### Comparison Between Patients With Retrocaput and Patients With Retrocollis

Eighteen patients (52.9%) had retrocaput, whereas the remaining 16 (47.1%) patients had retrocollis. We found that the age, gender, duration of symptoms, history of BTX-A injection, baseline Tsui score, the proportion of patients combined with other cervical movement patterns, and SRR were not different between the two groups (Table 1). The average dose of BTX-A seems to be higher in patients with retrocollis than in patients with retrocaput (281.3 ± 100.1 units vs. 241.7 ± 54.2 units). However, this difference was not statistically significant (*P* = 0.266). It was interesting to notice that more retrocollis patients combined dystonic movements in other areas of the body (62.5 vs. 16.7%, *P* = 0.006).

We then analyzed the injected muscles of the two subtypes (Table 2). We found that SPCa and SSCa were still the most frequently injected muscles in both groups. LS, SCM, and TPZ were commonly injected in both groups and the frequencies of injection were not different between the two groups. However, there were some differences that deserve our attention: (1) Although RCPM was mainly involved in head extension, its frequency of injection tended to be higher in patients with retrocollis (62.5 vs. 33.3%, *P* = 0.089). (2) In patients with retrocaput, only two patients received a low dose (18.8 ± 7.2 units) injection in ES at the T1–T4 level. However, four patients with retrocollis received a higher dose (79.7 ± 49.1 units) injection in ES at the T4–L5 level. (3) Although SSCe and SPCe were rarely injected, they were only injected in patients with retrocollis, but not in patients with retrocaput.

### Adverse Events

Adverse events (AE) were observed in 10 patients, among whom four reported dysphagia, four posterior cervical muscle weakness (PCMW), and two both dysphagia and PCMW. No patient reported dry mouth or local pain at the injection site. All these AE were mild and disappeared in ~2 weeks with no requirement for special treatment.

To determine the possible reason for the occurrence of AE, we reviewed the clinical features and the protocols of injection of these 10 patients (Table 3). We found that patients with AE tended to have a better SRR than those without AE (77.5 ± 15.9% vs. 65.4 ± 16.1%, *P* = 0.054). We speculate whether this may be due to the use of higher BTX-A doses in these patients, but contrary to our speculation, the doses used in these patients were even lower than those without AE (226.3 ± 45.8 units vs. 274.5 ± 88.0 units*, P* = 0.112).

**Table 3:** Clinical features of the 10 patients who showed adverse events.

**Number/** ** Gender**	**Age (years)**	**Duration of CD** ** (years)**	**Baseline Tsui Score**	**Dystonic** ** movement patterns**	**Dose of BTX-A (Units)**	**Protocol of injection**		**Subjective overall symptom relief rate**	**Adverse events**
						**Left (Units)**	**Right (Units)**		
**No.1** Male	51	8	7	Retrocaput	BOTOX 225	SPCa 50 SSCa 50 ES (T4) 12.5	SPCa 50 SSCa 50 ES (T4) 12.5	90%	Dysphagia
**No. 2** Male	49	9	6	Retrocaput + Torticaput + Limb dystonia	Hengli 200	SPCa 62.5 SSCa 37.5	SPCa 62.5 SSCa 37.5	70%	Dysphagia
**No. 3** Female	38	6	7	Retrocaput + Torticaput + No-no tremor	BOTOX 200	SPCa 62.5 SSCa 62.5	SPCa 25 SSCa 50	90%	Dysphagia
**No. 4** Female	60	6	4	Retrocaput	BOTOX 200	LS 25 SPCa 25 SSCa 25 RCPM 25	LS 25 SPCa 25 SSCa 25 RCPM 25	60%	Dysphagia
**No.5** Male	40	4	8	Retrocollis + Torticaput + Laterocaput	BOTOX 300	SCM 50 SPCa 25 SSCa 50 RCPM 12.5 RCPm 12.5	SCM 50 SPCa 25 SSCa 50 RCPM 12.5 RCPm 12.5	90%	Dysphagia PCMW
**No.6** Female	60	7	14	Retrocollis + Torticaput + Laterocollis + Limb dystonia	Hengli 287.5	LS 25 SPCa 37.5 SSCa 37.5 RCPM 25 RCPm 12.5	LS 25 SPCa 50 SSCa 37.5 RCPM 25 RCPm 12.5	80%	Dysphagia PCMW
**No.7** Male	58	7	7	Retrocollis + OMD	Hengli 250	LS 25 TPZ 37.5 SPCa 50 SSCa 25	LS 25 TPZ 0 SPCa 62.5 SSCa 25	50%	PCMW
**No. 8** Male	56	5	2	Retrocollis	Hengli 150	TPZ 25 SPCa12.5 SSCa 37.5	TPZ 25 SPCa 12.5 SSCa 37.5	100%	PCMW
**No. 9** Male	61	3	5	Retrocollis + MS	Hengli 200	SCM 12.5 SPCa 25 SSCa 50 RCPM 0 RCPm 12.5	SCM 12.5 SPCa 25 SSCa 50 RCPM 12.5 RCPm 0	65%	PCMW
**No. 10** Male	61	3	14	Retrocaput + Yes-yes tremor	Hengli 250	LS 12.5 SPCa 50 SSCa 25 RCPM 12.5	LS 25 SPCa 75 SSCa 25 RCPM 25	80%	PCMW

*SCM, sternocleidomastoid; SPCa, splenius capitis; SSCa, semispinalis capitis; TPZ, trapezius; LS, levator scapulae; RCPM, rectus capitis posterior major; RCPm, rectus capitis posterior minor; ES, erector spinae; OMD, oromandibular dystonia; MS, Meige syndrome; PCMW, posterior cervical muscle weakness*.

We then analyzed the protocol of injection applied to these patients. Bilateral SCM injection has long been considered a potential risk factor for dysphagia. However, of the six patients who had dysphagia, only one patient had received the bilateral injection in SCM. Out of the six patients who had PCMW, besides SPCa and SSCa, four patients received an injection in RCPM and/or RCPm.

## Discussion

The present study retrospectively analyzed the characteristics, target muscles, as well as the efficacy of BTX-A injection in patients with RCD. The frequency of RCD was 2.9% in the present investigation, which was lower than those reported previously. For example, Jost et al. reported a frequency of RCD in 7.5% of the patients with CD (3), and Papapetropoulos et al. established that 14.8% of the patients with CD had features of RCD (6). This discrepancy could be because we included only those patients with RCD as their most problematic feature.

Despite the inclusion only of the patients who had RCD as their most problematic feature, we still found a combination with other cervical movement patterns in a large proportion of the patients (61.8%). We established that the pure form of RCD occurred rarely, which was in accordance with a previous study (6). Similar to the findings of Papapetropoulos et al. (6), we also observed a combination of dystonic movements in other areas of the body in 38.2% of the patients, which included oromandibular dystonia, dystonia of the limb, dystonia of the trunk, and Meige syndrome.

Concerning the distribution of the target muscles, symmetrical participation of bilateral muscles is expected in RCD based on functional anatomy, and a bilateral injection scheme was also suggested by some researchers (12). Although most of our patients showed bilateral muscle activation, unilateral or asymmetric activation of cervical muscles also occurred. This finding could be explained by the existence of other cervical movement patterns.

Our study results suggested that SPCa and SSCa, the two biggest posterior cervical muscles, were the most commonly involved, and both of them were bilaterally injected in the majority of the patients. This result was consistent with the aforementioned view of Reichel (12). In the study of Papapetropoulos et al., a bilateral injection was also applied to SPCa in the majority of patients, but their frequency of injection for SSCa was much lower than that in our study (33/34 vs. 4/53) (6). Nevertheless, Jost et al. proposed that SSCa was a primary muscle but SPCa was a secondary muscle for retrocaput (3).

In addition to SPCa and SSCa, LS and TPZ were the commonly injected muscles in our study. Although the main function of LS is to elevate the shoulder, bilateral contraction of LS could retract the head and neck (13), and this action is facilitated by TPZ because the bilateral contraction of the TPZ pars descendens causes an extension of the head and neck (14). Bilateral injection of TPZ pars descendens has been suggested in several investigations (3, 6, 12), whereas bilateral LS injection was suggested in only one study (6).

Our study suggested that SCM was another commonly injected muscle in RCD. The study of Reichel also emphasized the role of bilateral SCM contraction in retrocaput (12). The effects of the simultaneous contraction of bilateral SCM can lead to both head flexion and extension, which depend on the state of contraction of the other muscles of the cervical spine: 1) if the cervical spine is rigid and rectilinear due to the contraction of the paravertebral muscles, the simultaneous contraction of bilateral SCM leads to flexion of the head; and 2) if the cervical spine is not fixed, this bilateral contraction results in extension of the head (15). Therefore, we need to carefully evaluate the specific posture of each individual patient with RCD to determine whether SCM participates in the head extension.

In addition to the aforementioned big muscles, suboccipital muscles also play an important role in RCD, which included RCPM, RCPm, and OCI. Our study found a significant involvement of RCPM (47.1%), and the majority (15/16) had the bilateral injection. However, only a small part (11.8%) of the patients had RCPm activation, which could be explained by the finding in a recent study that the primary function of RCPm was to stabilize the occipitoatlantal joint (16). Although OCI has been proposed as the primary muscle for retrocaput in some studies (3, 12), we only injected it in 8.8% of the patients. This could be because SPECT/CT revealed the activation of RCPM in patients with RCD, thus we performed more EMG detection and injection in RCPM than in OCI. For the injection of these three muscles, the spinous process of C2 is a very important landmark (17).

Based on the anatomy, RCPM contributed to retrocaput but not to retrocollis; however, we observed even more patients with retrocollis who received the injection in RCPM. A possible explanation could be that retrcollis was diagnosed by observing the abnormal angle between the cervical and thoracic spine, but it was difficult to judge whether the angle between the head and the neck was normal in these patients. With the activation of head extension muscles revealed by EMG in retrocollis patients, we considered that most retrocollis patients combined retrocaput, and pure retrocollis could hardly exist alone. This could also explain why patients with retrocaput and those with retrocollis had similar spectra of dystonic muscles.

Besides cervical muscles, we also found that 17.6% of the patients had ES activation. The reason why ES attracted our attention was that these patients had complaints of considerable local pain, and subsequent EMG detection confirmed their activation. The ES is composed not only of a single muscle but a group of muscles. They extend throughout the lumbar, thoracic, and cervical regions, and bilateral contraction of these muscles extends the spine (18). Our results showed that patients with retrocaput had only mild ES activation in the upper thoracic segment, whereas patients with retrocollis had obvious ES activation in the lower thoracic and lumbar segments. There are two possibilities for the ES activation: 1) the activation of ES itself can indeed participate in the retraction of the head and neck; and 2) the ES activation could be due to the spread of dystonic movements from the neck to the trunk, because we have noticed that three of the four retrocollis patients who had ES activation combined with obvious trunk extension. This finding supports the view of a previously published study which indicated that RCD may predict the spread of the dystonic movement to other regions of the body (19).

The total dose of BTX-A that we used was similar to the average dose used for all types of CD reported in a recent consensus guideline (20). However, the average dose applied for each muscle in our investigation was lower than that in the consensus guideline, which could be explained by the fact that the majority of the muscles in the present study were bilaterally injected. For the response to BTX-A injection, most of our patients showed satisfactory symptom relief: 55.9% of the patients had SRR ≥ 70%; in 35.3% of them, it was between 40 and 70%; and in 8.8%, it was <40%. This result seems to be better than the one obtained in the study of Papapetropoulos et al., which reported that 24.5% had excellent relief, 32.1% had moderate relief, 16.9% had mild relief, and 24.5% had no response (6). The reason for this discrepancy could be that we performed more extensive detection and applied the injection to a wider spectrum of target muscles.

The present study revealed that dysphagia (17.6%) and PCMW (17.6%) were the most frequently observed adverse events for patients with RCD. A recent review reported that the incidence of neck weakness in all types of patients with CD after BTX-A injection was 14% (21), which was slightly lower than our results. In the study of Papapetropoulos et al. in patients with RCD, no subjects showed PCMW (6). This could be explained by the fact that more posterior cervical muscles were injected in our study. The incidence of dysphagia reported in the review for all types of patients with CD was 11%, which was lower than ours (21), while another study reported a higher incidence of dysphagia (20%) (22).

The causes of dysphagia remained unclear, but the most accepted mechanism was the direct diffusion of the toxin into surrounding structures involved in the swallowing movement (23). Due to the proximity of the SCM to the pharyngeal muscles, the injection of SCM was discussed in the literature as a potential risk factor for BTX-A-associated dysphagia, especially bilateral injection of SCM (22). To determine the possible reason for the occurrence of dysphagia, we reviewed the clinical features and the protocols of injection for patients with AE. However, we found that the presence of dysphagia was not related to the demographics of the patients, BTX-A dose, or the injection scheme. This finding was in accordance with the ones of Kutschenko et al., who reported that the presence of dysphagia was not related to patient age or gender, BTX-A total dose, BTX-A dose in the SCM, or bilateral SCM injections (22). The study by Comella et al. also suggested that neither the total dose nor injection into particular muscles differed between those with dysphagia and those without (24). More studies should be conducted in terms of the mechanism for the presence of dysphagia following BTX-A injection.

There are several limitations of the present study: (1) the patients' data were only retrospectively analyzed and the study was not randomized; (2) the efficacy of BTX-A injection relied on subjective ratings rather than on validated scales; and (3) the EMG detections in the present study were performed without ultrasound guidance.

## Conclusion

In conclusion, the present study suggested that SPCa and SSCa were the two most commonly injected muscles in RCD. LS, TPZ, and SCM were also frequently injected. More attention should be paid to RCPM, RCPm, and ES. The majority of the target muscles in this study were bilaterally injected. Notably, most of the patients with RCD had satisfactory symptom relief by the administration of BTX-A.

## Data Availability Statement

The raw data supporting the conclusions of this article will be made available by the authors, without undue reservation.

## Ethics Statement

The studies involving human participants were reviewed and approved by Institutional Research Ethics Committee of Tongji Hospital affiliated to Tongji University. The patients/participants provided their written informed consent to participate in this study. Written informed consent was obtained from the individual(s) for the publication of any potentially identifiable images or data included in this article.

## Author Contributions

YH and LP: acquisition and analysis of the data. JS: statistical analysis and revision of the manuscript. SC: SPECT/CT scanning and analysis. YP and XZ: BTX-A injection. LJ: revision of the manuscript. FT: design of the study, analysis of the data, and drafting of the manuscript. All authors contributed to the article and approved the submitted version.

## Funding

This work was supported by the Medical Innovation Project of the Shanghai Science and Technology Commission (20Y11906000) and the Clinical Science and Technology Innovation Project of Shanghai Shen-Kang Hospital Development Center (SHDC12020119).

## Conflict of Interest

The authors declare that the research was conducted in the absence of any commercial or financial relationships that could be construed as a potential conflict of interest.

## Publisher's Note

All claims expressed in this article are solely those of the authors and do not necessarily represent those of their affiliated organizations, or those of the publisher, the editors and the reviewers. Any product that may be evaluated in this article, or claim that may be made by its manufacturer, is not guaranteed or endorsed by the publisher.
